# Molecular diversity of deep-sea fishes (Actinopterygii: Teleostei) in the western South Atlantic: A high diversity and new findings revealed by DNA barcoding

**DOI:** 10.1371/journal.pone.0347925

**Published:** 2026-07-14

**Authors:** Heloísa De Cia Caixeta, Claudio Oliveira, Marcelo Roberto Souto de Melo

**Affiliations:** 1 Instituto Oceanográfico da Universidade de São Paulo, São Paulo, Brazil; 2 Instituto de Biociências da Universidade Estadual Paulista ‘Júlio de Mesquita Filho’, Botucatu, Brazil; Universidade Federal do Rio de Janeiro, BRAZIL

## Abstract

The deep sea hosts approximately 15% of all fish species, but the difficulty of sampling limits our knowledge, causing large gaps in geographic distribution, and many deep-sea fish groups still require taxonomic revisions. Herein, the deep-sea fishes collected in the southern Brazil, western South Atlantic, are studied through DNA barcoding resulting in a better understanding of the biodiversity and genetic diversity of the region. The samples were obtained during oceanographic cruises aboard the Brazilian R/V Alpha Crucis, focusing on the continental slope off southern Brazil at depths of 200–1,500 meters. A total of 170 sequences of actinopterygian fishes from 102 different species of 49 families and 19 orders were generated. The DNA barcoding identified 84 sequences at the species level, with the remaining being identified at least to the genus level. A high level of genetic divergence between species was observed, ranging from 3.9% to 36.7%. We provided the first COI sequences for 13 species that, to date, were not represented in databases. Among the species already represented in online databases with COI sequences, we expanded the geographic coverage of existing data. As a result, we increased representation for 16 species in the Atlantic, 26 species in the South Atlantic, and 28 species in the western South Atlantic. Our efforts revealed a diverse deep-sea fish fauna in the region, highlighting new occurrences and demonstrating a high genetic divergence between some taxa.

## Introduction

The deep sea, defined as the ocean areas deeper than 200 m, comprises over 90 percent of the marine environment and encompasses diverse and extreme habitats, such as the continental slopes, pelagic habitats, canyons, trenches, and hydrothermal vents [1,2]. In recent years, important discoveries have been made about deep-sea fish diversity, including investigations of day-night community patterns by eDNA [3], diet [4], new records [5,6], and descriptions of new species using integrative taxonomy [7,8]. Nevertheless, only a small portion of these habitats has been explored, and many deep-sea species remain undescribed [9].

Since the proposal of DNA barcoding [10], several studies have used this technique as a standardized protocol that has advanced taxonomic studies for identifying and describing new species [8,11–13]. The survey of the deep-sea fish diversity using DNA barcoding has been applied to several regions, including the Mediterranean Sea [14], the North and South Pacific [15,16], the North Atlantic [17], and the Indian Ocean [18]. However, studies involving DNA barcoding of deep-sea fishes in the South Atlantic are scarce [15,19].

In a recent checklist, 712 deep-sea fish species from the Brazilian Economic Exclusive Zone (EEZ) were listed, including 5 Myxini, 87 Chondrichthyes, and 620 Actinopterygii [20]. Since then, taxonomic knowledge of Brazilian deep-sea actinopterygians has increased considerably, with assessments of biodiversity [21,22], new records [6,23], and new discoveries [8,24–26].

Nevertheless, even considering these studies, the use of molecular tools to identify deep-sea fish species in the Brazilian EEZ remains rare, limited to a few taxonomic groups [6,8,27]. Here, for the first time, a comprehensive study is dedicated to enhancing the understanding of the diversity of the western South Atlantic (WSA) deep-sea ray-finned fishes (Actinopterygii: Teleostei) by identifying the species through DNA barcoding.

## Materials and methods

### Taxa sampling

**Fig 1:**
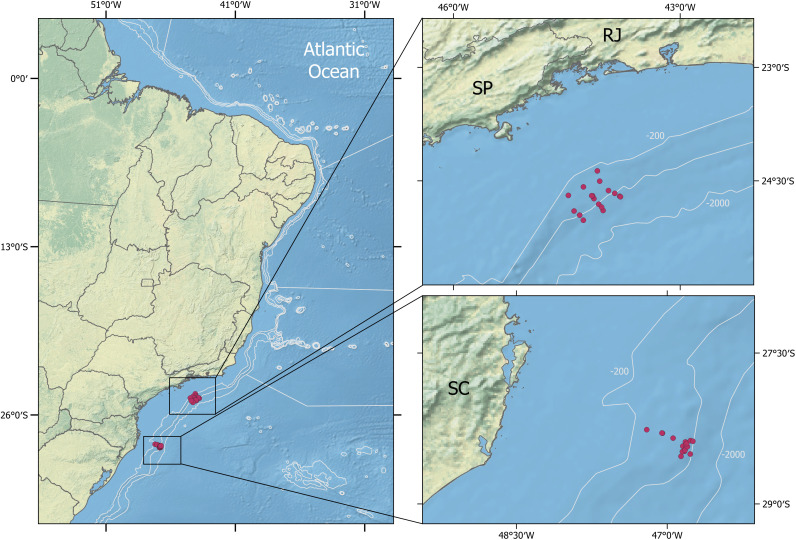
Map of the western South Atlantic, Brazilian margin, indicating the sampling areas (red circles). Sampling areas located off Ilhabela, São Paulo, Brazil (24°22’S– 25°01’S, 43°45’W–44°32’W) between 390 and 1,500 m; and off Florianopolis, Santa Catarina, Brazil (28°15’S–28°42’S, 46°44’W–47°12’W) between 274 and 1,232 **m.**

The specimens were collected during two oceanographic cruises onboard the Brazilian R/V *Alpha Crucis* using a semi-balloon bottom open trawl with 27 meters in the lower rope, mesh sizes of 100 mm in the wings and body, and 25 mm in the codend, and fish traps. The cruises were launched on the continental slope of southern Brazil off Ilhabela, São Paulo (24°22’S– 25°01’S, 43°45’W–44°32’W), between 390 and 1,500 m deep, and off Florianopolis, Santa Catarina (28°15’S–28°42’S, 46°44’W–47°12’W), between 274 and 1,232 m deep. Both cruises sampled at target depths in a range of 100 m between the minimum and maximum depth; however, due to the use of an open trawl, specimens may be collected during the release and recovery net operations. Thus, all depth mentions refer to the target sampling depths. The sample points are represented by red circles on a map produced in QGIS v. 3.24.2 [28] (Fig 1). Soon after the net recovery, the specimens were sorted and selected, and a musculature sample was removed from the right side. Each specimen received an identification number that matched the tissue sample. About 2,000 tissue samples were obtained and preserved in 96% ethanol, including 1,400 from actinopterygians.

The specimens were preserved in formalin and, in the laboratory, transferred to ethanol for collection preservation. The tissue samples are deposited at the Coleção Biológica Prof. Edmundo Nonato – COLBio.

Collection permits were issued by the Instituto Chico Mendes de Conservação da Biodiversidade (SISBIO permits #28054−4, 82624−1), Secretaria da Comissão Interministerial para Recursos do Mar da Marinha do Brasil (Portaria No. 223), and the Comitê de Ética em Uso de Animais em Pesquisa e Ensino do Instituto Oceanográfico da Universidade de São Paulo (CEUA permit #16 to MRSM).

### Laboratory procedures

The specimens were previously identified at the species level using taxonomic revisions and species keys [29,30]. The DNA was extracted using the Wizard Genomic DNA Purification (Promega Corp., WI, USA) and DNeasy Blood & Tissue Kit (Qiagen, USA) extraction kits, following the manufacturer’S protocols. Subsequently, the extracted DNA was quantified using a Nanodrop ND-1000 spectrophotometer. The mitochondrial cytochrome c oxidase subunit I (COI) gene was amplified and sequenced using universal primers (Table 1) [31,32].

**Table 1:** Universal primers used in this study.

Primer	Sequence 5’ - 3’
FISHF1	TCAACCAACCACAAAGACATTGGCAC
FISHR1	TAGACTTCTGGGTGGCCAAAGAATCA
FISHF2	TCGACTAATCATAAAGATATCGGCAC
FISHR2	ACTTCAGGGTGACCGAAGAATCAGAA
FISHF6	ACYAAYCACAAAGAYATTGGCA
FISHR7	TARACTTCTGGRTGDCCRAAGAAYCA

Universal primers were used to amplify and sequence the mitochondrial cytochrome c oxidase subunit I (COI) gene.

The Polymerase chain reaction (PCR) was performed using 12.5 µL of 2X GoTaq® Green Master Mix, 0.5 µL of each primer, 2 µL of DNA, and Nuclease–Free Water to a final volume of 25 µL. Fragments of the COI gene were amplified using an initial denaturation at 95°C for 3 minutes, followed by 30–35 cycles of amplification: denaturation at 94°C for 30 seconds, annealing at 50°C to 54°C for 45 seconds, and extension at 72°C for 1 minute. The final extension was at 72°C for 7 minutes. PCR products were visualized on a 1% agarose gel and purified with the SAP–Exo purification kit (Jena Bioscience, Jena, Germany). Bidirectional sequencing was performed using the BigDye Terminator v3.1 Cycle Sequencing kit on an ABI 3500 Series Genetic Analyzer sequencer (Applied Biosystems®) at the Instituto de Biotecnologia (IBTEC), Botucatu, São Paulo, Brazil

### Sequence identification and molecular data analysis

The chromatograms were edited and quality-checked, and the consensus sequences were generated in Geneious v. 4.8.5 [33]. Each sequence was verified by the Basic Local Alignment Search – BLASTn [34] and BOLD Barcoding ID [35] from a threshold of similarity of 98% with the sequences available on those databases.

All sequences were submitted to the BOLD (https://www.boldsystems.org/) database under the project “DEEP OCEAN: Diversity Ecology and Evolution of Deep-Sea Fishes” (code: DOP). The multiple alignment was performed in Geneious v. 4.8.5, and the nucleotide substitution model Kimura2-Parameters (K2P) was used to determine distances within taxonomic levels using MEGA software v11.0 [36].

A Maximum Likelihood (ML) phylogenetic tree was performed by the software RAxML v8.2.10 [37]. The best ML topology was found using the evolutionary model GTR + G, and node support was estimated from 1,000 bootstrap pseudoreplicates. The phylogenetic tree was visualized and edited using FigTree v. 1.4.4 (http://tree.bio.ed.ac.uk/software/figtree/). As outgroup, sequences of three chondrichthyans also collected during the cruises were used: *Scyliorhinus haeckelii* (Miranda Ribeiro, 1907) (DOP145−24); *Galeus mincaronei* Soto, 2001 (DOP146−24); and *Zameus squamulosus* (Günther, 1877) (DOP147−24). The taxonomic classification adopted herein follows Betancur et al. [38], except for *Lopholatilus villari* Miranda Ribeiro, 1915, which follows Fricke et al. [39].

For the species with unclear similarity results, i.e., those for which the threshold considered matched more than one species, the results were further investigated using all database sequences for each genus using both the maximum likelihood tree and genetic divergence. In these cases, the multiple alignment was performed using the WSA sequences, database sequences from BOLD Systems and GenBank [35,40] of congeners, and sequences from the closest genus and family as outgroups. The alignment was submitted to the online tool ElimDupes (https://www.hiv.lanl.gov/content/sequence/elimdupesv2/elimdupes.html) to compare sequences and remove duplicates, thereby reducing computational time for analysis. The intraspecific and interspecific K2P distances were calculated for each genus. Sequence groups were considered the same taxon if the distance within the group was < 2% [41]. The Maximum Likelihood tree by genus was performed using the evolutionary model GTR + G and 1,000 bootstrap pseudoreplicates. Therefore, species identification was performed using results from similarity searches, K2P distances, and ML phylogenetic trees by genus.

## Results

A total of 107 Actinopterygii species were collected, and tissues for molecular analysis were removed from each. Sequences were produced for 102 species. The following five species were identified using taxonomic keys [29,42], but the sequences did not have a good quality (HQ = 0–14%): the beryciform *Sio nordenskjoldii* (Lönnberg, 1905), and the gadiforms *Gadella imberbis* (Vaillant, 1888); *Gadomus capensis* (Gilchrist & von Bonde, 1924); *Hymenocephalus billsam* Marshall & Iwamoto, 1973; and *Ventrifossa macropogon* Marshall, 1973.

**Fig 2:**
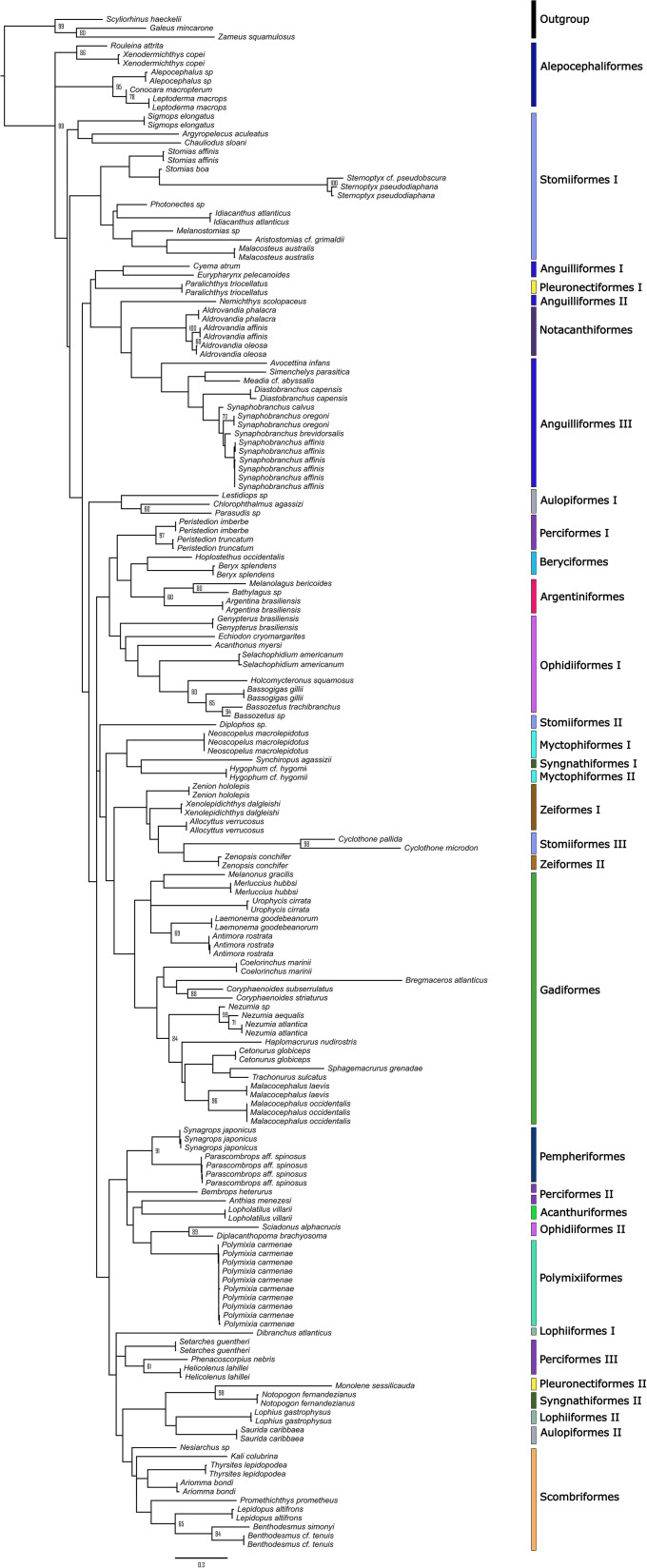
Phylogenetic tree based on COI sequences obtained from 170 vouchers of 102 species. Topology recovered by Maximum Likelihood inference and bootstrap values as node support values; those below 60% are hidden.

A total of 170 individuals from 102 different species from 87 genera, 49 families, and 19 orders were sequenced for the COI gene (Table 2). The length of sequences varies between 306 and 650 base pairs (bp), resulting in a multiple alignment matrix of ~651 bp. Only eight out of the 170 sequences are short, with a length of up to 475 bp. The ML tree recovered all genera, except *Stomias*, 90% of the families, and 47% of the orders as monophyletic (Fig 2). All species are clearly delimited in the topology, especially those identified only at the genus level.

**Table 2:** List of species identified from 170 sequenced vouchers (excluded here three vouchers regarding the outgroup sequences).

Order	Family	Species	BOLD Accession number	Sequence status
Acanthuriformes	Latilidae	*Lopholatilus villarii*	DOP143−24	
			DOP144−24	
Alepocephaliformes	Alepochephalidae	*Alepocephalus* sp.	DOP149−24	SAO
			DOP150−24	
		*Conocara macropterum*	DOP001–24	WSA
			DOP002–24	
		*Leptoderma macrops*	DOP151−24	
			DOP152−24	
		*Rouleina attrita*	DOP003–24	WSA
		*Xenodermichthys copei*	DOP004–24	WSA
			DOP005–24	
Anguilliformes	Cyematidae	*Cyema atrum*	DOP153−24	SAO
	Nemichthyidae	*Avocettina infans*	DOP154−24	SAO
		*Nemichthys scolopaceus*	DOP155−24	SAO
	Synaphobranchidae	*Meadia* cf. *abyssalis*	DOP006–24	AO
		*Diastobranchus capensis*	DOP156−24	AO
			DOP157−24	
		*Synaphobranchus affinis*	DOP007–24	WSA
			DOP008–24	
			DOP009–24	
			DOP010–24	
			DOP011–24	
			DOP012–24	
		*Synaphobranchus brevidorsalis*	DOP013–24	WSA
		*Synaphobranchus calvus*	DOP014–24	AO
		*Synaphobranchus oregoni*	DOP015–24	WSA
			DOP016–24	
		*Simenchelys parasitica*	DOP017–24	WSA
	Eurypharyngidae	*Eurypharynx pelecanoides*	DOP018–24	WSA
Argentiniformes	Argentinidae	*Argentina brasiliensis*	DOP019–24	
			DOP020–24	
	Bathylagidae	*Melanolagus bericoides*	DOP021–24	AO
		*Bathylagus* sp.	DOP022–24	
Aulopiformes	Chlorophitalmidae	*Chlorophthalmus agassizi*	DOP158−24	SAO
		*Parasudis* sp.	DOP159−24	SAO
	Paralepididae	*Lestidiops* sp.	DOP160−24	First sequence of species
	Synodontidae	*Saurida caribbaea*	DOP023–24	SAO
			DOP024−24	
Beryciformes	Berycidae	*Beryx splendens*	DOP025−24	WSA
			DOP026−24	
	Trachichthydae	*Hoplostethus occidentalis*	DOP027−24	SAO
Gadiformes	Bregmacerotidae	*Bregmaceros atlanticus*	DOP161−24	WSA
	Macrouridae	*Cetonurus globiceps*	DOP028−24	AO
			DOP029−24	
		*Coelorinchus marinii*	DOP030−24	
			DOP031−24	
		*Coryphaenoides striaturus*	DOP032−24	New occurrence/ AO
		*Coryphaenoides subserrulatus*	DOP033−24	New occurrence/ AO
		*Haplomacrourus nudirostris*	DOP034−24	AO
		*Malacocephalus laevis*	DOP162−24	SAO
			DOP163−24	
		*Malacocephalus occidentalis*	DOP035−24	WSA
			DOP164−24	
			DOP165−24	
		*Melanonus gracilis*	DOP166−24	AO
		*Nezumia aequalis*	DOP036−24	AO
		*Nezumia atlantica*	DOP037−24	First sequence of species
			DOP038−24	
		*Nezumia* sp.	DOP167−24	First sequence of species
		*Sphagemacrurus grenadae*	DOP039−24	
		*Trachonurus sulcatus*	DOP168−24	
	Merluccidae	*Merluccius hubbsi*	DOP040−24	
			DOP041−24	
	Moridae	*Antimora rostrata*	DOP042−24	WSA
			DOP043−24	
		*Laemonema goodebeanorum*	DOP044−24	AO
			DOP148−24	
	Phycidae	*Urophycis cirrata*	DOP045−24	
			DOP046−24	
Lophiiformes	Lophiidae	*Lophius gastrophysus*	DOP047−24	
			DOP048−24	
	Ogcocephalidae	*Dibranchus atlanticus*	DOP049−24	SAO
Myctophiformes	Neoscopelidae	*Neoscopelus macrolepidotus*	DOP050−24	WSA
			DOP051−24	
			DOP052−24	
	Myctophidae	*Hygophum* cf. *hygomii*	DOP053−24	WSA
			DOP054−24	
Notacanthiformes	Halosauridae	*Aldrovandia affinis*	DOP055−24	SAO
			DOP056−24	
		*Aldrovandia phalacra*	DOP057−24	SAO
			DOP058−24	
		*Aldrovandia oleosa*	DOP059−24	SAO
			DOP060−24	
Ophidiiformes	Bythitidae	*Sciadonus alphacrucis*	DOP061−24	First sequence of species
		*Diplacanthopoma brachyosoma*	DOP169−24	SAO
	Carapidae	*Echiodon cryomargarites*	DOP062−24	AO
	Ophidiidae	*Bassogigas gillii*	DOP063−24	AO
			DOP064−24	
		*Bassozetus sp.*	DOP065−24	AO
		*Bassozetus trachibranchus*	DOP066−24	SAO
		*Genypterus brasiliensis*	DOP067−24	
			DOP068−24	
		*Holcomycteronus squamosus*	DOP069−24	AO
		*Selachophidium americanum*	DOP071−24	First sequence of species
			DOP072−24	
	Acanthonidae	*Acanthonus myersi*	DOP073−24	AO
Pempheriformes	Acropomatidae	*Parascombrops* aff. *spinosus*	DOP074−24	First sequence of species
			DOP075−24	
			DOP076−24	
			DOP077−24	
		*Synagrops japonicus*	DOP078−24	WSA
			DOP079−24	
			DOP080−24	
Perciformes	Bembropidae	*Bembrops heterurus*	DOP081−24	First sequence of species
	Peristediidae	*Peristedion truncatum*	DOP082−24	SAO
			DOP083−24	
		*Peristedion imberbe*	DOP084−24	First sequence of species
			DOP085−24	
	Serranidae	*Anthias menezesi*	DOP170−24	
	Setarchidae	*Setarches guentheri*	DOP086−24	SAO
			DOP087−24	
	Scorpaenidae	*Helicolenus lahillei*	DOP088−24	
			DOP089−24	
		*Phenacoscorpius nebris*	DOP171−24	
Pleuronectiformes	Bothidae	*Monolene sessilicauda*	DOP090−24	SAO
	Paralichthydae	*Paralychthys triocellatus*	DOP091−24	First sequence of species
			DOP092−24	
Polymixiiformes	Polymixiidae	*Polymixia carmenae*	DOP093−24	
			DOP094−24	
			DOP095−24	
			DOP096−24	
			DOP097−24	
			DOP098−24	
			DOP099−24	
			DOP100−24	
			DOP101−24	
			DOP102−24	
Scombriformes	Ariommatidae	*Ariomma bondi*	DOP103−24	SAO
			DOP104−24	
	Chiasmodontidae	*Kali colubrina*	DOP105−24	WSA
	Trichiuridae	*Benthodesmus* cf. *tenuis*	DOP106−24	WSA
			DOP107−24	
		*Benthodesmus simonyi*	DOP108−24	WSA
		*Lepidopus altifrons*	DOP109−24	WSA
			DOP110−24	
	Gempylidae	*Nesiarchus* sp.	DOP111−24	SAO
		*Promethichthys prometheus*	DOP172−24	SAO
		*Thyrsites lepidopodea*	DOP112−24	First sequence of species
			DOP113−24	
Stomiiformes	Gonostomatidae	*Cyclothone microdon*	DOP114−24	WSA
		*Cyclothone pallida*	DOP115−24	WSA
		*Diplophos* sp.	DOP116−24	First sequence of species
		*Sigmops elongatus*	DOP117−24	WSA
			DOP118−24	
	Sternoptychidae	*Argyropelecus aculeatus*	DOP119−24	WSA
		*Sternoptyx pseudodiaphana*	DOP120−24	SAO
			DOP121−24	
		*Sternoptyx* cf. *pseudobscura*	DOP173−24	
	Stomiidae	*Aristostomias* cf. *grimaldii*	DOP122−24	First sequence of species
		*Chauliodus sloani*	DOP123−24	WSA
		*Idiacanthus atlanticus*	DOP124−24	WSA
			DOP125−24	
		*Malacosteus australis*	DOP126−24	SAO
			DOP127−24	
		*Melanostomias* *melanops*	DOP128−24	SAO
		*Photonectes* sp.	DOP129−24	Putative new species/ first sequence of species
		*Stomias affinis*	DOP130−24	SAO
			DOP131−24	
		*Stomias boa*	DOP132−24	WSA
Syngnathiformes	Callionymidae	*Synchiropus agassizii*	DOP174−24	SAO
	Centriscidae	*Notopogon fernandezianus*	DOP133−24	
			DOP134−24	
Zeiformes	Grammicolepididae	*Xenolepidichthys dalgleishi*	DOP135−24	WSA
			DOP136−24	
	Oreosomatidae	*Allocyttus verrucosus*	DOP137−24	
			DOP138−24	
	Zeidae	*Zenopsis conchifer*	DOP139−24	
			DOP140−24	
	Zeniontidae	*Zenion hololepis*	DOP141−24	WSA
			DOP142−24	

List of species identified from 170 sequenced vouchers (excluded here three vouchers regarding the outgroup sequences), BOLD accession numbers, and COI sequence status indicating the first sequences for the Atlantic Ocean (AO), South Atlantic Ocean (SAO), and western South Atlantic (WSA), the first sequences for species, putative new species and new occurrences.

**Fig 3:**
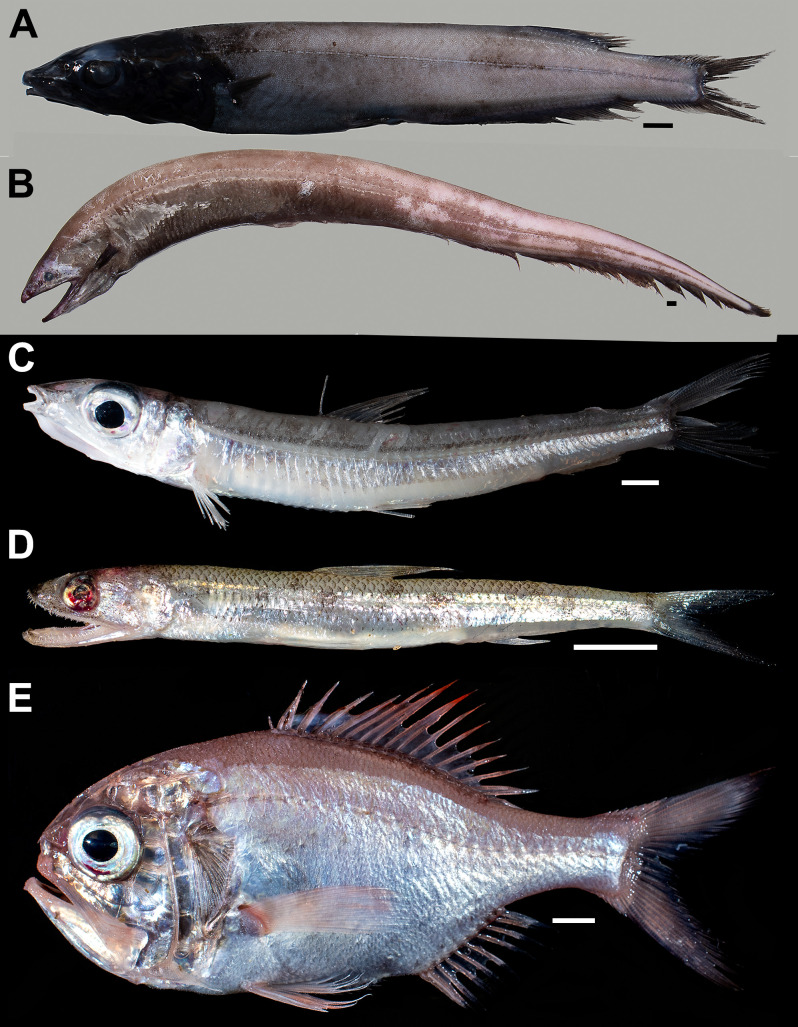
Representative orders of deep-sea fishes collected on the southern Brazilian continental slope. **(A)** *Conocara macropterum* (Alepocephaliformes: Alepocephalidae), targeted depth of 1,190 m; **(B)** *Synaphobranchus* *brevidorsalis* (Anguiliformes: Synaphobranchidae), targeted depth of 1,450 m; **(C)** *Argentina brasiliensis* (Argentiniformes: Argentinidae), targeted depth of 393 m; **(D)** *Saurida caribbaea* (Aulopiformes: Synodontidae), targeted depth of 161 m; **(E)** *Hoplostethus occidentalis* (Beryciformes: Trachichthydae), targeted depth of 500 **m.** Scale bar = 1 cm.

**Fig 4:**
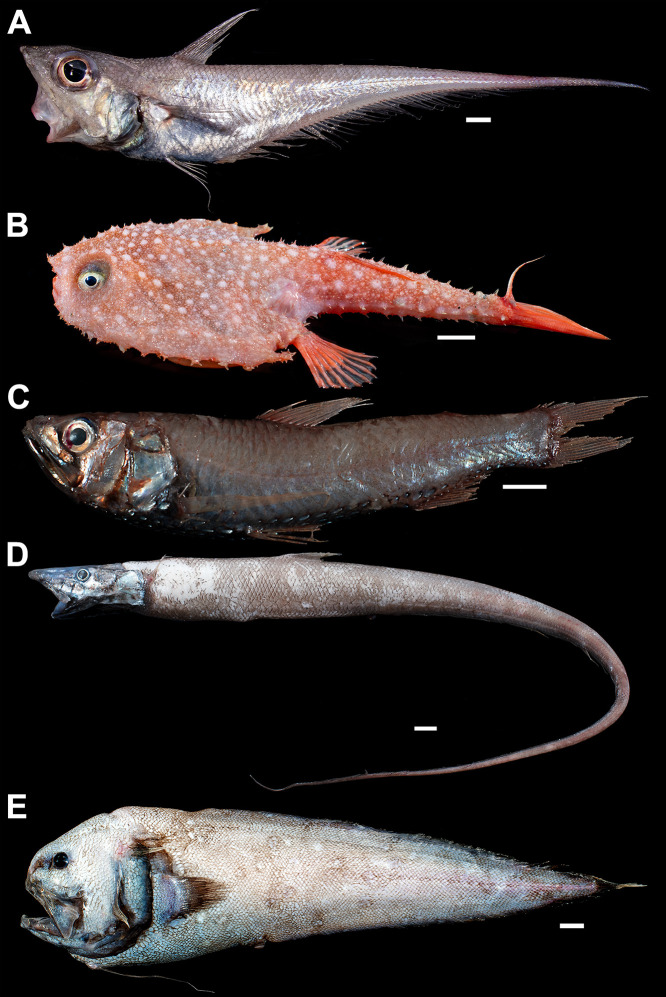
Representative orders of deep-sea fishes collected on the southern Brazilian continental slope. **(A)** *Coelorinchus marinii* (Gadiformes: Macrouridae), 800 m deep; **(B)** *Dibranchus atlanticus* (Lophiiformes: Ogcocephalidae), targeted depth of 393 m; **(C)** *Neoscopelus macrolepidotus* (Myctophiformes: Neoscopelidae), targeted depth of 794 m; **(D)** *Aldrovandia affinis* (Notacanthiformes: Halosauridae), targeted depth of 900 m; and **(E)** *Acanthonus myersi* (Ophidiiformes: Ophidiidae), targeted depth of 1,342 **m.** Scale bar = 1 cm.

**Fig 5:**
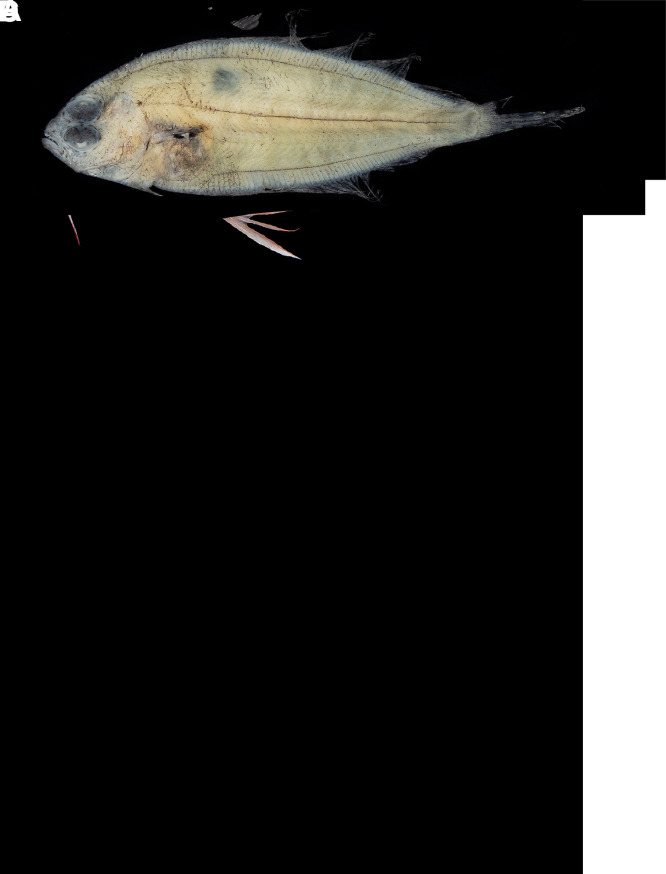
Representative orders of deep-sea fishes collected on the southern Brazilian continental slope. **(A)** *Helicolenus lahillei* (Perciformes: Sebastidae), targeted depth of 504 m; **(B)** *Synagrops japonicus* (Phemperiformes: Acropomatidae), targeted depth of 603 m; **(C)** *Monolene sessilicauda* (Pleuronectiformes: Bothidae), targeted depth of 400 m; **(D)** *Polymixia carmenae* (Polymixiiformes: Polymixiidae), targeted depth of 400 **m.** Scale bar = 1 cm.

**Fig 6:**
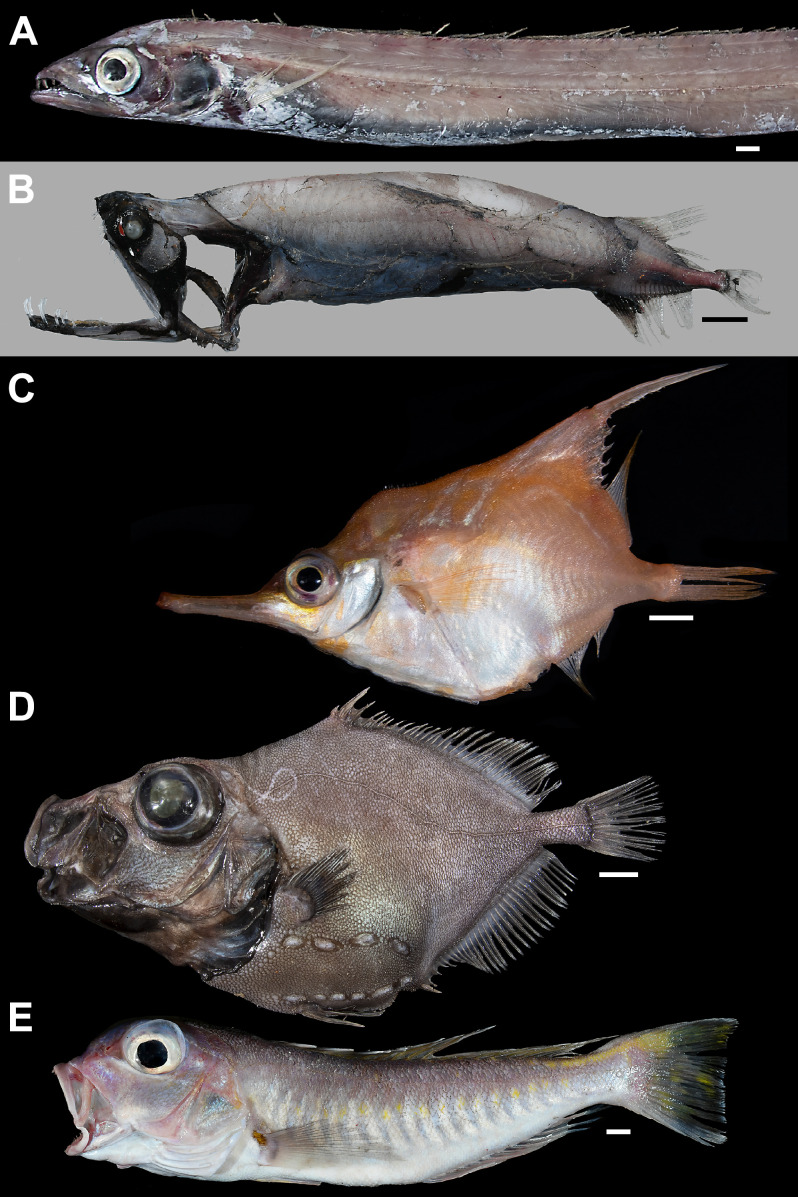
Representative orders of deep-sea fishes collected on the southern Brazilian continental slope. **(A)** *Lepidopus altifrons* (Scombriformes: Trichiuridae), anterior portion, targeted depth of 400 m; **(B)** *Aristostomias* cf. *grimaldii* (Stomiiformes: Stomidae), targeted depth of 737 m; **(C)** *Notopogon fernandezianus* (Syngnathiformes: Centriscidae), targeted depth of 504 m; **(D)** *Allocyttus verrucosus* (Zeiformes: Oreosomatidae), targeted depth of 1,190 m; **(E)** *Lopholatilus villarii* (Acanthuriformes: Malacanthidae), targeted depth of 400 **m.** Scale bar = 1 cm.

**Fig 7:**
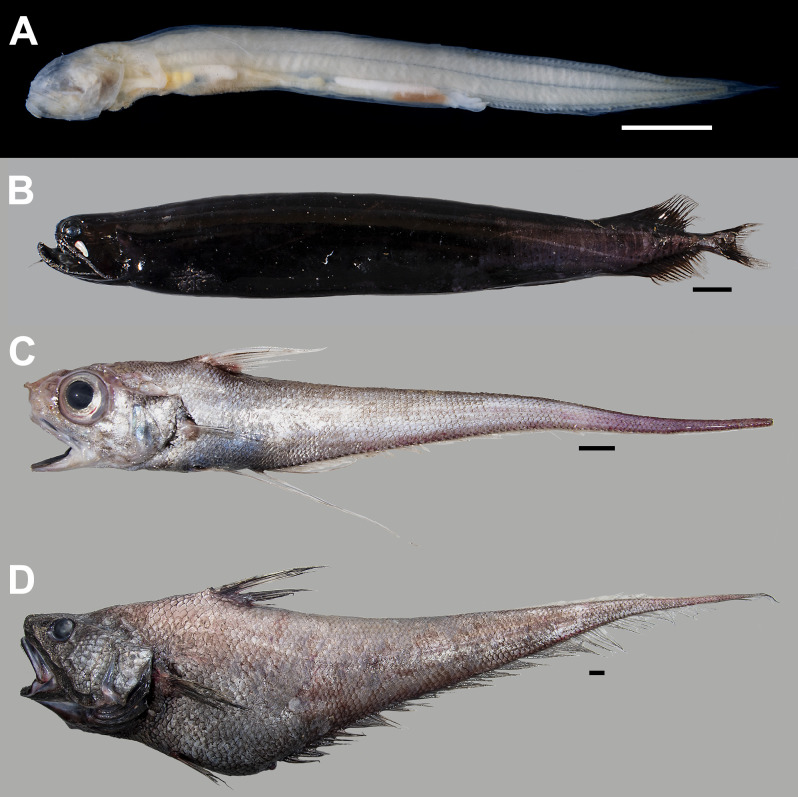
Described species, new records, and putative new species in the Brazilian Economic Exclusive Zone: **(A)** *Sciadonus alphacrucis* (Ophidiiformes: Bythitidae) targeted depth of 794 m; **(B)** *Photonectes* sp.(Somiiformes: Stomiidae) targeted depth of 1,190 m; **(C)** *Coryphaenoides striaturus* (Gadiformes: Macrouridae) targeted depth of 1,200 m; **(D)** *Coryphaenoides subserrulatus* (Gadiformes: Macrouridae) targeted depth of 900 **m.** Scale bar = 1 cm.

**Fig 8:**
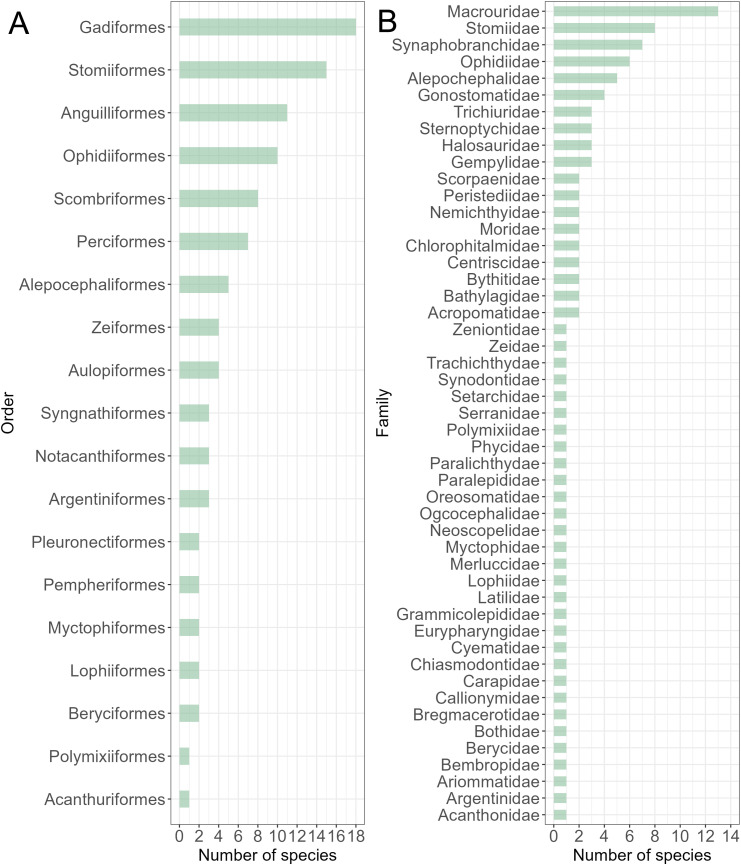
Number of species sequenced within each taxonomic level: order (A) and family(B).

Among the 19 orders sequenced (Figs 3–7), the most diverse is Gadiformes, followed by Stomiiformes and Anguilliformes, with 18, 15, and 11 species, respectively. Two orders are represented by a single species each, Acanthuriformes and Polymixiiformes (Fig 8A). The most diverse families are the gadiform Macrouridae, the stomiiform Stomiidae, and the ophidiiform Ophidiidae, with 13, 8, and 6 species, respectively. Thirty families are represented by a single species each (Fig 8B).

About 75% of the species analyzed have a similarity above 98% with GenBank sequences, and 86% with BOLD sequences (S1 Table). The COI barcoding results identified 84 of the 102 deep-sea actinopterygians at the species level and were mostly consistent with the preliminary morphological identifications. BLASTn results showed good query coverage, with 95% of the identifications having query coverage above 75% and most searches around 100%.

As expected, the mean K2P genetic divergence increased with increasing taxonomic levels, from 0.4% at the species level to 14.7% at the order level. The average K2P distance within each taxonomic level is 0.4% ± 0 for the species, 2.4% ± 0.3 for the genera, 7.9% ± 1.2 for the families, and 14.7% ± 1.6 for the orders (Table 3). The range of K2P between the species is 3.9% to 36.7%; between the genera, 9.9% to 36.1%; between the families, 13.9% to 35.4%; and between the orders, 19.2% to 29.5% (S2–S5 Tables). The following six orders were represented by two species, each with a high genetic distance between species: Beryciformes (D_K2P_ = 17%), Lophiiformes (D_K2P_=24%), Myctophiiformes (D_K2P_=22%), Pempheriformes (D_K2P_=15%), Pleuronectiformes (D_K2P_ = 25%), and Syngnathiformes (D_K2P_=26%). Ten orders were represented by three to 18 species, wherein Notacanthiformes provided the lowest genetic divergence between *Aldrovandia affinis* and *Aldrovandia oleosa* (D_K2P_ = 3.9%).

**Table 3:** Summary of genetic divergence (K2P) at each taxonomic level.

Nucleotide	Taxa	Min divergence	Max divergence	Mean divergence	SE divergence
Within species	102	0	0.029	0.004	0
Within genus	87	0	0.218	0.024	0.003
Within family	48	0	0.245	0.079	0.012
Within order	19	0	0.253	0.147	0.016

Summary (minimum, maximum, mean, and standard error) of genetic divergence (K2P) at each taxonomic level (species, genus, family, and order in a hierarchical order).

## Discussion

### Deep-sea fish diversity from the western South Atlantic

While DNA barcoding has been extensively used to assess the diversity and taxonomy of deep-sea fish worldwide, regions such as the Brazilian EEZ remain largely underexplored [13,14,16,17]. The present work successfully identified a high diversity of deep-sea fishes from the Brazilian margin.

A total of 102 species of deep-sea actinopterygians were identified from 170 COI sequences, of which 84 species (82%) were previously reported in the Brazilian EEZ. Ten of those species were originally described from southern Brazil: *Argentina brasiliensis* Kobyliansky, 2004; *Bembrops heterurus* (Miranda Ribeiro, 1903); *Genypeterus brasiliensis* Regan, 1903; *Kali colubrina* Melo, 2008; *Lophius gastrophysus* Miranda Ribeiro, 1915; *Lopholatilus villarii* Miranda Ribeiro, 1915; *Paralichthys triocellatus* Miranda Ribeiro, 1903; *Sciadonus alphacrucis* Melo, Gomes, Møller & Nielsen, 2021; *Synaphobranchus calvus* Melo, 2007; and *Polymixia carmenae* Caixeta, Oliveira & Melo, 2024 [8,24,39,43–48]. In addition, nine species sequenced are endemic to southern Brazil or also occur in nearby areas in Uruguay and Argentina: *Anthias menezesi* Anderson & Heemstra, 1980; *Argentina brasiliensis* Kobyliansky, 2004; *Coelorinchus marinii* Hubbs, 1934; *Genypterus brasiliensis* Regan, 1903; *Lopholatilus villarii* Miranda Ribeiro, 1915; *Merluccius hubbsi* Marini, 1933*; Polymixia carmenae* Caixeta, Oliveira & Melo, 2024; *Sciadonus alphacrucis* Melo, Gomes, Møller & Nielsen, 2021; and *Synaphobranchus calvus* Melo, 2007.

The findings include two species recently described, the bythitid *Sciadonus alphacricus* Melo, Gomes, Møller & Nielsen, 2021, described by Melo et al. [24] and subsequently barcoded by dos Reis Júnior et al. [6], and the polymixiid *Polymixia carmenae* Caixeta, Oliveira & Melo, 2024 [8], which was described based on morphology (morphometrics, meristics, osteology) and molecular characters (COI, 12S, 16S), in addition to one putative new stomiid species is under investigation, assigned to the genus *Photonectes*. (Fig 7B). The genus *Alepocephalus* urges for  a thorough taxonomic review and *Alepocephalus* sp. can be a new species that deserves further investigation. There are three new records in the Brazilian continental slope, the macrourids *Coryphaenoides striaturus* Barnard, 1925, and *C. subserrulatus* Makushok, 1976 [49] (Fig 7C, 7D), and the beryciform *Sio nordenskjoldii* – interestingly, the three species are widely spread in the southern hemisphere, but *C. subserrulatus* was previously known from Uruguay and Argentina, *S. nordenskjoldii* from Uruguay, and *C. striaturus* from the Rio Grande Plateau [42,50–58].

Even though deep-sea zones are remote and difficult to access, many deep-sea species are subject to anthropogenic impacts from fishing and marine pollution. [59–61]. Indeed, five barcoded species are targets for the Brazilian commercial fisheries fleet, including the tilefish *Lopholatilus villarii*, the Argentinean hake *Merluccius hubbsi*, the blackfin goosefish *Lophius gastrophysus*, the pink cusk-eel *Genypterus brasiliensis*, and the silvery John dory *Zenopsis conchifer* (Lowe, 1852) [62,63]. According to the Brazilian official endangered species list [64], 16 deep-sea fish species are at risk of extinction, including *Lopholatilus villarii,* which is listed as Vulnerable.

Considering species already represented in online databases, we include sequences for 16 species that have sequences only from the Southern, Indian, or Pacific Oceans. Additionally, 26 species had sequences from the North Atlantic but not from the South Atlantic, and another 28 species had sequences from the eastern South Atlantic. In summary, a total of 70 species had the sequence coverage expanded to the WSA.

The COI sequences of nine species were not previously represented in any online database: the gadiform *Nezumia atlantica* (Parr, 1946); the ophidiiforms *Selachophidium americanum* (Nielsen, 1971) and *Sciadonus alphacrucis*; the pempheriform *Parascombrops* aff. *spinosus* (Schultz, 1940); the perciforms *Bembrops heterurus* and *Peristedion imberbe* Poey, 1861; the pleuronectiform *Paralichthys triocellatus* Miranda Ribeiro, 1903; the scombriform *Thyrsites lepidopodea* Lesson, 1831; and the stomiiform *Aristostomias cf. grimaldii* Zugmayer, 1913.

We could not identify sequences at the species level for four taxa: the aulopiform *Lestidiops* sp., the gadiform *Nezumia* sp., and the stomiiforms *Photonectes* sp. and *Diplophos* sp. These sequences do not match any sequences in the online databases with a similarity above 98%. For *Lestidiops* sp. and *Nezumia* sp., we were also unable to identify them to the species level using taxonomic keys. These results highlighted the effect of the absence of comparative sequences in online databases on species identification by DNA barcoding. In addition, the challenge of identifying it using both taxonomic keys and COI sequences can provide insights, such as the need for taxonomic revisions and the identification of putative new species, as in the case of *Photonectes* sp. The morphological characteristics of the specimen differ from those of other species in the genus, and combined with the genetic divergence, we consider it a putative new species.

The sequence of the gonostomatid *Diplophos* sp. has a similarity of 94% with sequences of *Diplophos australis* Ozawa, Oda & Ida, 1990 (S1 Table). Examining the specimen, we supposed that it could be a representative of the genus *Manducus,* however, the specimen is not in good condition because it was cut in half during collection, and only the anterior half was recovered, damaging the key morphological characteristics for identification, such as the skin, body photophores, and the relative position of dorsal and anal fins. Compared with the sequences of *Manducus maderensis* (Johnson 1890) available online, our sequence differs by 21.5%.

Among the bothids, the single species sequenced was identified as *Monolene sessilicauda* Goode, 1880, with 98% similarity to *M. sessilicauda* from Canada and Florida, the western North Atlantic, and the Gulf of Mexico, respectively. Another congener reported from Brazilian waters, *M. antillarum* Norman, 1933, has similarities in pigmentation, meristics, and morphometric data, suggesting the need of a comprehensive taxonomic revision of *Monolene* occurring in the western Atlantic [65].

According to Ward [41], individuals showing less than 2% genetic divergence in COI are more likely to belong to the same species, whereas those with greater divergence are more likely to be distinct species. Among the generated sequences, the mean distances within a species (0.4%) are congruent with those of intraspecific values. The mean genetic divergence within the genera, families, and orders is 2.4%, 7.9%, and 14.7%, respectively. Ward [41] also explored the divergences at those supraspecific taxonomic levels, finding values considerably higher than ours; however, the correspondence between genetic divergence and the taxonomic limits of genus, family, and order is not straightforward.

The barcoding tree generated by ML inference recovered 90% of the families and 47% of the orders as monophyletic. In addition, bootstrap values are below 60% in most clades that group orders and families, being higher in terminal branches. This reflects the effectiveness of the COI gene in identifying species, as discussed previously, and in recovering hypotheses of genus and family relationships. However, the use of the COI gene to resolve deeper phylogenies (*i.e.*, order, class) is limited [12,32], resulting in a high number of polytomies and less efficient recovery of monophyletic clades.

### Are online databases useful to identify deep-sea fishes?

Worldwide, there are many challenges to scientifically exploring the deep sea, including the need for an oceanographic vessel, the availability of technology (e.g., ROVs, HOVs, and deep-water nets), and the expensive operational costs [66,67]. In the context of better understanding Brazilian deep-sea biodiversity, even with these challenges, there was a significant increase in data from the 2000s onwards especially after the cruises of the French RV Thalassa (1999, 2000), the Brazilian RV Atlântico Sul (1996–1999) and the Brazilian FVs Diadorim and Solancy Moura (1996–2002), Brazilian OSV Astro Garoupa (2001, 2007), the USA RVs Luke Thomas (2009) and Seward Johnson (2011), the French R/V Antea (2015–present), and the Braziilan RV Alpha Crucis (2019, 2020) [20].

However, beyond the economic scope, the recent challenges in studying Brazilian deep-sea fishes are also due to the limited or no availability of tissues for molecular analyses. It was only in the past few years that tissue samples became available due to the recent expeditions, such as those conducted onboard the RV *Alpha Crucis*. As a result, the number of COI sequences available in online databases is limited, especially for endemic species. Such a paucity of samples and sequences poses many challenges for the use of DNA barcoding to properly identify WSA deep-sea fishes.

When analyzing the WSA sequences generated during this study for molecular species identification, we encounter several challenges: unrepresentative taxon coverage in online databases, unclear similarity results, and sequences that do not correspond to the actual taxa, likely due to misidentifications. S1 Table illustrates instances in which the closest sequences detected by BLASTn and the BOLD Barcoding ID resulted in unclear species identification, which could only be verified by reviewing the specimen morphologically, by further genetic distances, and by comparing all database sequences from each genus. Results from similarity analysis are efficient when the queried species is represented in the database; however, if it is not, these methods can yield a false identification [68].

In 34 cases, the similarity results showed that more than one species matched the generated sequences (>98%). For some cases, the identifications were then confirmed morphologically by examining museum specimens or checking original descriptions, such as *Phenacoscorpius nebris*, *Anthias menezesi,* and *Argyropelecus aculeatus*, while others need to be further investigated with all database sequences, *i.e.*, not only the sequences presented in the similarity result, but by maximum likelihood tree and distance methods for each genus, including *Rouleina attrita*, *Sphagemacrurus grenadae*, and *Merluccius hubbsi* (S1–S3 Figs; S6–S8 Tables). A review of the literature indicates that most studies over the past decade have used tree topology for other analyses, with likelihood and Bayesian inference gaining strength, even though neighbor-joining remains the most widely used [12]. Additionally, in some cases, distance-based methods for species identification may not adequately represent species relationships and underestimate the potential of DNA barcoding [12].

Incongruences were found in two commercially important species, *Merluccius hubbsi* and *Lopholatilus villarii*, but for different reasons. The sequences of the Argentinean hake *M. hubbsi*, matched those also identified as *M. hubbsi*, but also with the North Pacific hake *M. productus* (Ayres, 1855), and the Southern hake *M. australis* (Hutton, 1872). The sequence of *M. productus* (Genbank accession number GU324172.1) was produced from a sample commercially purchased from the Pacific Ocean, and the sequences of *M. australis* (BOLD accession numbers EHPQ015–19 and EHPQ016–19) are from Argentina. However, since our samples were obtained from the southern Atlantic Ocean and the morphological identifications indicate *M. hubbsi*, in addition to most sequences with a similarity of 99–100% being of *M. hubbsi,* and finally the maximum likelihood recovered our sequences in the *M. hubbsi* clade, we understand that as two distinct cases of identification error in the online databases.

Another case returns more than one species of the same genus, the tilefish *Lopholatilus villarii* and the great northern tilefish *Lopholatilus chamaeleonticeps* Goode & Bean, 1879. Our sequence matched those of both *L. villarri* from the South Atlantic and L. chamaeleonticeps from the United States and Mexico. Sequences of the two valid species of *Lopholatilus* are available online, with an interspecific distance of 1.3%, below the 2% threshold commonly used to delimit species. Overall, the COI gene successfully delineates species; however, rates of evolution vary across taxa and, in some cases, such as the youngest species and synonyms, make it difficult to recognize species limits [10,41]. Furthermore, integrating distance-based and character-based analyses, as well as morphology, might reveal a synonym for the two species.

Similarly, *Allocyttus verrucosus* showed high similarity with A. verrucosus from the South Atlantic, Pacific, and Indian Oceans, and with *A. folletti* Myers, 1960, but also with *Neocyttus helgae* (Holt & Byrne, 1908), *N. psilorhynchus* Yearsley & Last, 1998, and *N. rhomboidalis* Gilchrist, 1906. Those genera and species are morphologically very distinctive and therefore difficult to confuse [69], but the oreosmomatids exhibit low genetic divergence and are difficult to separate using DNA barcoding [41].

A remarkable case is *Helicolenus lahillei* Norman, 1937 (Fig 5C), which has a similarity index >98% (98.03–99.84%) with the sequences available of seven out of nine valid species of the genus: *H. avius* Abe and Eschmeyer, 1972; *H. barathri* (Hector, 1875); *H. dactylopterus* (Delaroche, 1809); *H. hilgendorfii* (Döderlein, 1884); *H. lahillei* Norman, 1937; *H. lengerichi* Norman, 1937; and *H. percoides* (Richardson & Solander, 1842). Eschmeyer (1969) questioned the validity of *H. hilgendorfii* and *H. lahillei* based on the morphological similarity*,* referring to the latter as a subspecies of *H. dactylopterus*. This seems to be a similar case to the scorpionfish *Setarches guentheri* Johnson, 1862, which was hypothesized to comprise five nominal species, but all of which were synonymized based on morphological and genetic evidence [70]. In this case, we suppose it might be the same, widespread species that needs taxonomic revision.

Another challenging case was identifying synaphobranchid eels. Seven species were sequenced: *Diastobranchus capensis* Barnard, 1923; *Meadia cf. abyssalis* (Kamohara, 1938); *Synaphobranchus affinis* Günther, 1877; *S. brevidorsalis* Günther, 1887; *S. calvus; S. oregoni* Castle, 1960; and *Symenchelis parasitica* Gill, 1879. The sequences avaibable on-line were assigned to four of the seven valid species of *Synaphobranchus*, however, the genus still proved difficult to identify using DNA barcoding methods. Both BLASTn and BOLD Barcoding ID results show more than one species with similarity above 98%. In our results, the sequences of *Synaphobranchus affinis* showed similarity with some sequences identified online as *Synaphobranchus kaupii* Johnson, 1862 and *Synaphobranchus brevidorsalis*; *S. calvus*, with *S. brevidorsalis* and *Diastobranchus capensis*; and *S. oregoni*, also with *S. kaupii*. Notably, the species of *Synaphobranchus* are extremely difficult to identify [30,71–73], leading to possible misidentifications or the existence of multiple cryptic species.

The similarity results can be misinterpreted because the closest sequence match depends on the data’s reliability [74]. Here, we can identify a possible misidentification in the sequence of *Bembrops heterurus* (Miranda Ribeiro, 1903) in databases based on morphological identification and the type species locality of our sequenced specimen. Another study focused on DNA barcoding of deep-sea fish has also identified misidentifications in databases [75].

Erroneous sequence identification can explain the observed increase in genetic divergence among fish [41]. For instance, *Bembrops heterurus* does not match any conspecific sequences in online databases, indicating a distance greater than 5%. However, our specimen was collected near its type locality [46], whereas the sequence available does not include the locality for the conference. Therefore, based on morphological identification and type locality, we believe that the online database sequence represents a misidentification.

On the other hand, the DNA barcoding was extremely useful for the identification of four species that could not be promptly achieved by morphology: the macrourid *Coryphaenoides striaturus*; two species of bristlemouths that are extremely similar, *Cyclothone microdon* Günther, 1878, and *C. pallida* Brauer, 1902; and the trichiurid *Benthodesmus simonyi* (Steindachner, 1891). For those cases, the molecular identification was later confirmed morphologically using the original descriptions and/or taxonomic revisions.

## Conclusions

Besides reporting a high diversity of deep-sea fishes from the western South Atlantic, we provide the first sequences of species in online databases, putative new species, and new occurrences for the Brazilian EEZ and the western South Atlantic. DNA barcoding techniques applied in this study reveal the high potential for accessing biodiversity, but also pose challenges in identifying species and in trusting primary results from BLASTn and BOLD Barcoding ID.

Even then, in most identifications (66%), similarity searches return clear results. DNA barcoding is an effective and powerful technique; however, we emphasize the importance of analyzing and exploring all available sequences in online databases for each species, rather than relying solely on the initial similarity results. In addition, identifying specimens by DNA barcoding is only possible and viable due to the existence of public database sequences; however, the conference and frequent revision of these sequences are crucial.

## Supporting information

S1 Table Results of similarity (%) and values of query cover (%) from BLASTn and BOLD ID. (XLSX)

S2 Table Genetic distances (K2P) between species. (XLSX)

S3 Table Genetic distances (K2P) between genera. (XLSX)

S4 Table Genetic distances (K2P) between families. (XLS)

S5 Table Genetic distances (K2P) between orders. (XLSX)

S6 Table Genetic distances (K2P) between species of the genus *Rouleina.* (XLSX)

S7 Table Genetic distances (K2P) between species of the genus *Sphagemacrurus.* (XLSX)

S8 Table Genetic distances (K2P) between species of the genus *Merluccius.* (XLSX)

S1 Fig Topology recovered by Maximum Likelihood inference of the genus *Rouleina*, bootstrap values as node support values, scale bar indicates 0.02 substitutions per site. (JPG)

S2 Fig Topology recovered by Maximum Likelihood inference of the genus *Sphagemacrurus*, bootstrap values as node support values, scale bar indicates 0.05 substitutions per site. (JPG)

S3 Fig Topology recovered by Maximum Likelihood inference of the genus *Merluccius*, bootstrap values as node support values, scale bar indicates 0.04 substitutions per site. (JPG)
