# Supplementary figures and images for: Molecular diversity of deep-sea fishes (Actinopterygii: Teleostei) in the western South Atlantic: A high diversity and new findings revealed by DNA barcoding
Source: PLoS One. 2026 Jul 14;21(7):e0347925. doi: 10.1371/journal.pone.0347925 (PMC13367907; doi:10.1371/journal.pone.0347925)

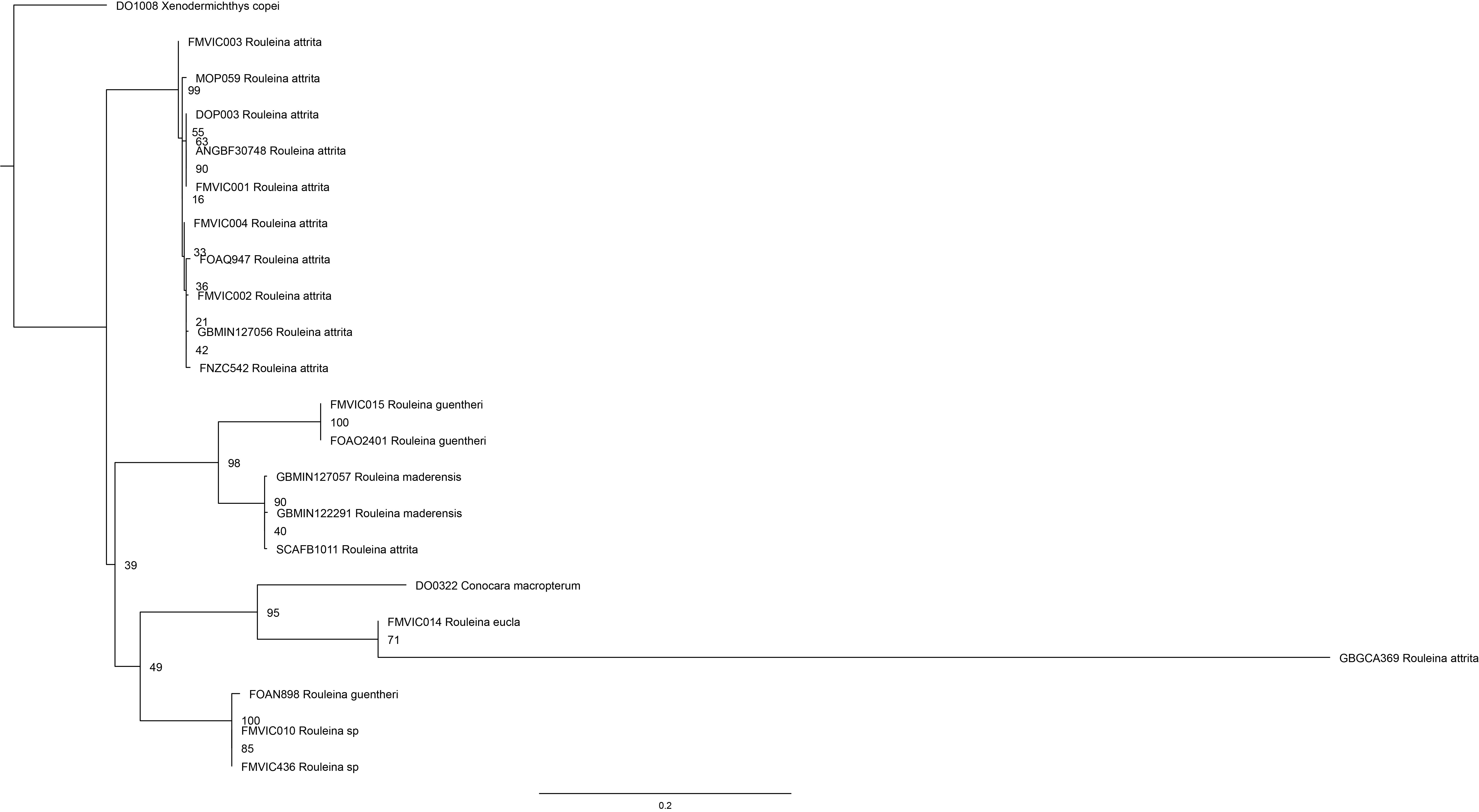

Supplement: S1 Fig — (JPG) [file pone.0347925.s009.jpg]

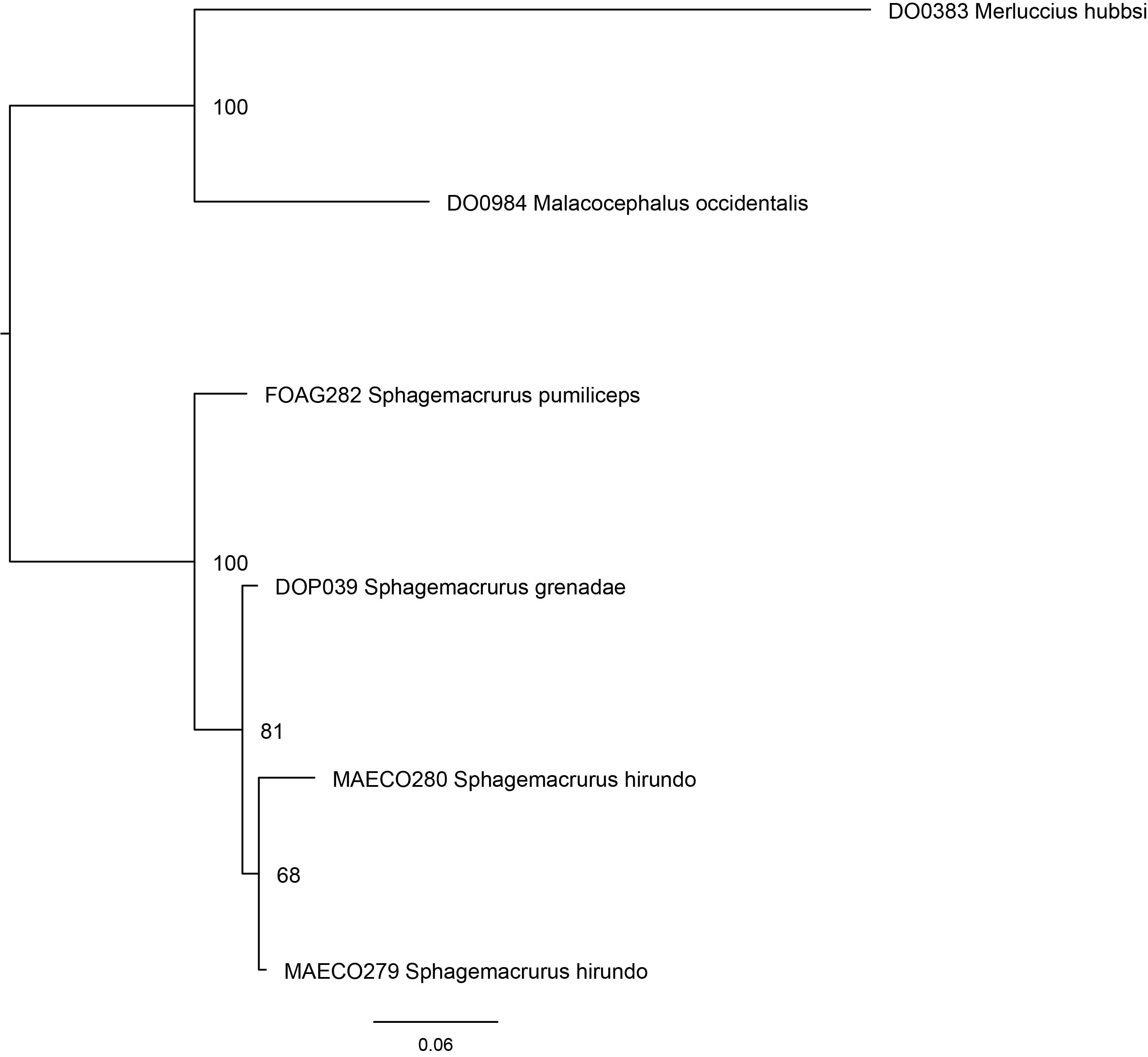

Supplement: S2 Fig — (JPG) [file pone.0347925.s010.jpg]
